# Risk Factors for Overweight and Obesity in Later School-Aged Children: Focus on Lifestyle Behaviours and Psychosocial Characteristics

**DOI:** 10.3390/healthcare12090912

**Published:** 2024-04-27

**Authors:** Yong-Sook Eo, Myo-Sung Kim

**Affiliations:** 1 College of Nursing, Dongguk University-WISE, Gyeongju 38066, Republic of Korea; nursingeo@dongguk.ac.kr; 2 Department of Nursing, Dong-Eui University, Busan 47340, Republic of Korea

**Keywords:** body esteem, childhood, health behaviour, obesity, overweight, personality

## Abstract

The study analysed the 12th wave (2019) of data from the Panel Study on Korean Children conducted by the Korea Institute of Child Care and Education. A total of 1174 children were selected as the subjects of the study. The results concerning the identifying factors influencing overweight and obesity in later school-aged children revealed that when compared to boys, girls were 1.66 times more likely to be overweight or obese. Moreover, for every one-hour increase in media usage time, the likelihood of being overweight or obese increased by 1.23 times, and for every one-point increase in body dissatisfaction, the probability of being overweight or obese increased by 2.07 times. However, among personality traits, neuroticism was associated with a 0.96 times lower likelihood of being overweight or obese. These findings underscore the significance of considering not only lifestyle factors but also psychosocial characteristics such as body dissatisfaction and neuroticism as predictive factors for overweight and obesity in later school-aged children, providing a basis for intervention.

## 1. Introduction

The prevalence of childhood obesity is steadily increasing worldwide, affecting nearly one in three children (29% of boys and 27% of girls) in the WHO European Region [1]. In Korea, the obesity rate among adolescents has increased 2.4 times from 5.5% in 2011 to 13.3% in 2021 [2]. According to the National School Health Examination (NSHE) conducted on 6–18-year-olds, the prevalence of overweight increased by 1.3 times from 6.6% in 2007 to 8.7% in 2017, and obesity increased by 1.7 times from 8.7% in 2007 to 15.0% in 2017 [3]. Childhood obesity is higher in later school age (including ages 10–12) than in early school age (including ages 6–9) [4,5], and the prevalence of overweight in school-aged children is higher than in adolescents [4,6].

Childhood obesity tends to persist into adulthood [7]. Additionally, obesity is associated not only with physical comorbidities such as asthma, cardiovascular metabolic diseases, sleep apnea, and dental caries [8,9] but also with mental health problems such as depression, Attention Deficit Hyperactivity Disorder (ADHD), eating disorders, school adjustment problems, bullying, and low self-esteem [10]. The WHO has stated that obesity is a strong risk factor for chronic diseases such as diabetes and cardiovascular diseases and increases the risk of complications from COVID-19 [1]. Therefore, it is essential to prevent and treat childhood obesity as it often leads to obesity in adulthood [11].

Obesity is known to be influenced by a complex interplay of environmental factors such as socioeconomic conditions and lifestyle factors, including physical activity and dietary habits [10]. Numerous studies have addressed the relationship between lifestyle behaviours and obesity [12,13,14]. It has been found that insufficient physical activity [15], the consumption of unhealthy foods including potatoes, meat, and sugary drinks [16], sedentary behaviour due to increased media usage [17], and insufficient sleep [18,19] are associated with a higher prevalence of overweight and obesity. These unhealthy lifestyle behaviours have been further exacerbated during the COVID-19 pandemic due to restrictions on outdoor activities, increased screen time, and changes in sleep patterns [12,13,14]. Furthermore, while environmental factors influence obesity, it is important to recognise inherent risk factors such as genetic predispositions and family history [20]. Genetics indeed play a pivotal role in health issues, including obesity [21].

Psychosocial factors are also noteworthy in understanding the factors related to childhood obesity [22]. Obesity is not merely a medical condition threatening physical health due to excessive weight but is heavily intertwined with societal and cultural perceptions and attitudes, exerting a significant impact on psychosocial aspects [23]. Childhood obesity carries risk factors for psychological and social issues such as bullying, social stigma, and peer rejection [24]. While these psychosocial problems have primarily been discussed as consequences of obesity [24,25], it is important to recognise them as cyclic and reciprocal factors contributing to the development of obesity, as individuals may avoid exercising in public spaces due to social stigma, leading to decreased physical activity levels and increased likelihood of obesity through repeated dieting and binge-eating cycles [26]. Therefore, examining psychosocial problems in childhood as factors influencing obesity and early intervention to address these issues are important in preventing overweight and obesity.

Body image is identified as a significant psychosocial predictor of childhood and adolescent obesity [26,27,28,29]. Children in late school age, where secondary sexual characteristics emerge, tend to be more concerned about their body image. Perceiving oneself as obese may lead to social impairment and psychological distress due to associated stressors [26,29,30]. Obese children tend to have a negative perception of their bodies compared to normal-weight children [31] and exhibit diminished self-concept, leading to higher academic stress [26].

There is growing evidence of the association between children’s personality traits and obesity [32]. Impulsivity among personality traits has been found to increase the risk of obesity [33]. Impulsive individuals may disrupt dietary habits and are susceptible to behaviours that offer immediate rewards or satisfaction, potentially leading to unhealthy eating habits. Associations have been reported between low conscientiousness and childhood obesity [32], higher openness to experience and lower conscientiousness in overweight and obese men, and lower neuroticism and openness to experience and higher agreeableness in overweight and obese women [34]. However, obesity has shown inconsistent associations across a wide range of personality domains, and there is a lack of research investigating the relationship between late school-aged children and personality traits. Therefore, understanding children’s personality traits related to obesity may facilitate more efficient management of childhood obesity.

The prevalence of overweight and obesity in Korean children is higher than in adolescents and pre-school children [4,5]; however, there is a lack of research on the predictors of obesity in late school-aged children. Thus, it is necessary to study a more comprehensive range of children with excess weight, including those who may progress to obesity, among late school-aged children.

## 2. Materials and Methods

### 2.1. Study Design

This study utilised a secondary analysis design aiming to identify risk factors related to obesity using data from the 12th wave of the Panel Study on Korean Children (PSKC) conducted by the Korea Institute of Child Care and Education (KICCE) in 2019 [35].

### 2.2. Participants and Ethical Consideration

The PSKC is a longitudinal study tracking 2150 infants born in 2008 from medical institutions nationwide until 2027. The sampling design utilised a two-stage stratified sampling method to enhance the representativeness of the sample, as the status of newborns born in hospitals closely resembled the overall population of newborns (minimising non-coverage error). In the first stage, nationwide obstetrics and gynecology hospitals were selected as primary sampling units using the number of deliveries as a measure of size, enabling probability proportional to size sampling. In the second stage, 30 newborn households were sampled from each selected medical institution as the sampling unit, to select newborn households born during the panel recruitment period as the sample. The survey, conducted annually, involves children, parents, and teachers. Initially, basic information, such as contact details, is confirmed through telephone interviews with the panel participants. Subsequently, survey questionnaires are sent by mail to the participants, followed by face-to-face interviews, observations, and performance tests conducted by surveyors using Tablet-PC Aided Personal Interviewing (TAPI).

The 12th wave of data pertains to children at the age of 11, corresponding to fifth graders in elementary school, representing the late school-age period. Out of a total of 1412 panel children, 1373 children participated after excluding those who refused due to reasons such as death, immigration, or long-term tracking failure, accounting for 98.9% of the sample. The survey was conducted from 17 July to 30 December 2019. Particularly, from the 12th wave onwards, psychological and social variables such as body esteem and personality, measured by the children themselves, have been included, allowing for an understanding of the relationship between children’s health status and their psychological and social characteristics.

**Figure 1:**
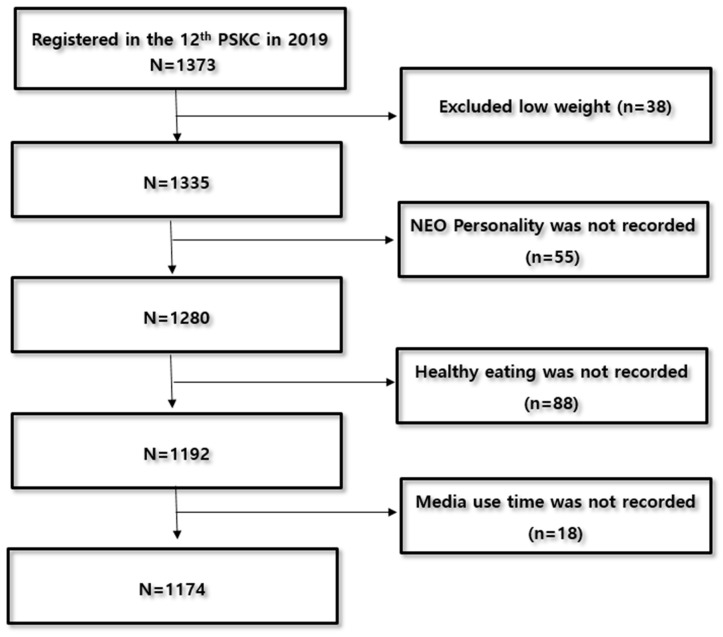
Flow chart of participants. Source: PSKC = Panel study on Korean children.

The data with personally identifiable information removed were downloaded from the PSKC website [http://panel.kicce.re.kr (27 January 2019)]. Among the total of 1373 participants, 38 children classified as underweight based on BMI were excluded. After excluding the missing data for variables such as the NEO personality inventory, dietary habits, and media usage time, a total of 1174 children were included in the analysis (see Figure 1). Each participant provided informed consent to the PSKC survey team before their inclusion in the survey (IRB No.: KICCEIRB-2019-01).

### 2.3. Measures

#### 2.3.1. Obesity

Obesity was assessed by calculating the body mass index (BMI) using the child’s weight and height and then determining the percentile based on the 2017 standard growth curves for children and adolescents. Trained interviewers who visited households directly measured the children’s body size, including height and weight. Those falling between the 5th and less than the 85th percentile were categorised as having a normal weight, those between the 85th and less than the 95th percentile were classified as overweight, and those at or above the 95th percentile were considered obese. This method is widely recognised and recommended by health organisations such as the World Health Organisation (WHO) and the Centers for Disease Control and Prevention (CDC) for assessing weight status in children and adolescents. The BMI calculation provides a standardised measure that accounts for differences in height and weight across age groups. Additionally, the use of percentile categories, as defined by the standard growth curves, allows for the classification of weight status relative to the child’s age and sex [36].

#### 2.3.2. Demographic Characteristics

The demographic characteristics of the subjects were analysed based on gender, perceived socioeconomic status, and health status. Gender was categorised as “male” and “female”. Subjective economic status is measured by a relative deprivation index regarding the family’s circumstances, consisting of items asking, “On a scale of 1 (very poorly off) to 10 (very well off), where do you think your family stands?”. Higher scores indicate a higher perceived economic level by the child. This item is used in the Comprehensive Survey of Children and Adolescents conducted by the Ministry of Health and Welfare in South Korea, serving as an indicator to measure the household circumstances of children aged 9–17 [37]. Health status is determined by the parents’ perception of their child’s health, assessed through the question, “How do you rate your child’s health?” [37]. Responses were recorded on a 5-point Likert scale, ranging from ‘very unhealthy (1 point)’, ‘unhealthy (2 points)’, ‘neither unhealthy nor unhealthy (3 points)’, ‘healthy (4 points)’ to ‘very healthy (5 points)’, with higher scores indicating better health. This method has been utilised in various studies [38,39] and was adopted for use in this study.

#### 2.3.3. Lifestyle Behaviours

Lifestyle behaviour variables included healthy eating, physical activity, media usage time, and sleep duration. These tools (except physical activity) are validated through a rigorous process involving experts in measuring the health behaviours of Korean Youth Risk Bahavior Web-based Survey (below KYRBS), overseen by the government [2]. Numerous studies utilising these data have been published in reputable international journals, validating the reliability of these tools [15,27]. These data were collected through questions asked to the child’s parents. The dietary assessment tool was developed based on items related to diet from the KYRBS conducted by the Korea Centers for Disease Control and Prevention [40]. It consists of 10 items related to the child’s diet, including consumption of carbonated beverages, instant foods, frequency of meals with family, and picky eating. Items were rated on a 3-point Likert scale ranging from ‘somewhat true (1 point)’, ‘neither true nor true (2 point)’ to ‘not true (3 point)’, with higher scores indicating unhealthy eating habits.

Physical activity consists of vigorous or moderate-intensity physical activities such as table tennis, swimming, brisk walking, excluding walking to and from school, for at least 30 min indoors and outdoors in one day within a week, except walking to and from school. Physical activity tools were modified based on the Korea National Health and Nutrition Examination [41]. It is composed of items asking about days with such physical activity for at least 30 min each week, and these are coded as follows: ‘not at all, 0 point’ ‘1–2 days a week, 1 point’, ‘3 or more days a week, 2 point’. Media usage time consists of questions about the time the child spends using TV, computer, mobile phone, and so on. The average weekly media usage time was calculated for both weekdays and weekends. Sleep time consists of questions asking about the time the child goes to bad and wakes up, recalling the most typical weekday during the semester. Daily sleep time was calculated by calculating the wake-up time and bedtime.

#### 2.3.4. Psychosocial Characteristics

Psychosocial characteristics were assessed using body esteem and the NEO Personality Inventory. The examination of body esteem utilised items adapted from the Body-Esteem Scale (BSE) developed by Mendelson and White [42], translated and modified by KICCE [32]. Five items were used, including four from the original scale assessing appearance satisfaction and body respect, with an additional item related to height, reviewed by experts. The tool’s validity was confirmed through KICCE peer mentoring and expert advisory meetings, followed by a pilot study for finalisation. The confirmed items included: ‘I am satisfied with my appearance’, ‘I am dissatisfied with my weight (reverse scored)’, ‘I like the way I look in the mirror’, ‘I would like to change many aspects of my appearance if I could (reverse scored)’, and ‘I am satisfied with my height (additional item)’. Although originally scored on a 4-point Likert scale ranging from ‘not at all true (1 point)’ to ‘very true (4 points)’, the scores were reverse-coded in this study, with higher scores indicating lower physical self-esteem. The internal consistency reliability (Cronbach’s α) of the questionnaire was 0.691, which, according to conventional standards, falls within the moderate range. This suggests that while the scale demonstrates a degree of consistency in measuring body esteem, there may be some variability in responses to individual items.

The NEO Personality Assessment System employed a validated and standardised test tool developed by Ahn and Ahn [43], adapted from Costa and McCrae’s NEO Personality Inventory (NEO-PI) [44], to measure child personality within Korean culture. This tool consists of 147 items, including 2 reliability-measuring items, organised into five factors and 18 sub-scales: neuroticism (anxiety, hostility, depression, impulsiveness, social withdrawal, and emotional distress), extraversion (sociability, dominance, and stimulation-seeking), openness (creativity, emotionality, and flexibility), agreeableness (warmth, trust, and tolerance), and conscientiousness (competence, organisation, and responsibility). Neuroticism reflects emotional instability or maladaptation, characterised by poor impulse control and ineffective stress management [44]. Extraversion denotes the level of psychophysical energy, while openness indicates high imagination, creativity, abundant ideas, and emotional richness [43]. Agreeableness represents a propensity for interpersonal relationships, characterised by trust, forming close relationships, and willingness to help others [44]. Conscientiousness refers to confidence, systematic and organised work, and a strong sense of responsibility [43]. Higher scores on neuroticism suggest greater emotional instability and susceptibility to stressors, while higher scores on extraversion indicate a more sociable and energetic personality. Higher scores on openness signify a greater propensity for creativity, imagination, and emotional depth. Similarly, higher scores on agreeableness suggest a more trusting, empathetic, and cooperative nature, whereas higher scores on conscientiousness reflect greater organisation, responsibility, and diligence. The assessment was conducted online via the Insight Psychological Assessment Research Institute’s survey page [http://inpsyt.co.kr] during face-to-face interviews, with a test duration of approximately 40 min. Scores were provided in T-scores, indicating the position of the child’s personality relative to the entire Korean child population.

### 2.4. Procedure and Statistical Analysis

Data collection for the 12th wave of the Panel Study on Korean Children took place from 17 July to 30 December 2019. To enhance the safety of the survey, the highly portable and accessible TAPI (for children and caregivers) was used. Furthermore, with over 90% of the previous surveyors secured, tailored group training based on individual surveyor experience was conducted. Self-report surveys were conducted through face-to-face interviews with children and parents, and the NEO Personality Inventory, a performance test, was administered online.

Data were analysed using the SPSS 26.0 (IBM Corp., Armonk, NY, USA). Descriptive statistics and frequency analyses were conducted to assess the general characteristics, lifestyle behaviours, and psychosocial characteristics of the subjects. Differences in demographic and lifestyle characteristics between normal-weight and overweight/obese children were analysed using the χ^2^-test or *t*-test. Additionally, multiple binary logistic regression was conducted to analyse the impact of key variables on overweight and obesity, using normal-weight children as the reference group and overweight/obese children as the comparison group. The adequacy of the logistic regression model was assessed using the −2 Log Likelihood (1243.223), Chi-square (χ^2^ =58.133, *p* < 0.001), and Hosmer–Lemeshow test (χ^2^ =7.660, *p* = 0.467), indicating a good fit for the model. The effect sizes of variables influencing the model were as follows: gender 0.201, media use time 0.174, body esteem 0.279, and neuroticism 0.189.

## 3. Results

### 3.1. Child Characteristics

Of the surveyed children, 13.9% were overweight and 10.4% were obese, totalling 24.3% being overweight or obese. Among the general characteristics, 51.9% were male and 48.1% were female. The subjective economic status and health status were rated at 6.92 points and 4.19 points, respectively. Healthy eating, physical activity, daily media usage time, weekday sleep duration, body esteem, and NEO personality traits are summarised in Table 1.

### 3.2. Difference in Overweight and Obesity According to Child Characteristics

There were differences in the degree of overweight and obesity according to child characteristics such as gender (χ2 = 9.955, *p* = 0.002), media usage time (t = −3.330, *p* = 0.001), body esteem (t = −4.322, *p* < 0.001), and extraversion personality traits (t = 2.181, *p* = 0.029). Specifically, females had a higher prevalence of overweight and obesity compared to males. Furthermore, overweight and obese children had longer daily media usage time, lower body esteem, and lower extraversion compared to children with normal weight (see Table 2). We have carefully reviewed the data and confirmed that there are no significant differences between the overweight and obese groups.

### 3.3. Predictors of Overweight and Obesity in Children

To identify predictors of overweight and obesity in children, we compared the influence of various factors using children with normal weight as the reference group. The results showed statistically significant differences in gender, media usage time, body esteem, and neuroticism. Females had a 1.66 times higher probability of being overweight or obese compared to males (OR = 1.66, 95% CI: 1.24, 2.21), and for each additional hour of media usage, the probability of being overweight or obese increased by 1.23 times (OR = 1.23, 95% CI: 1.07, 1.41). An increase of 1 point in body esteem was associated with a 2.07 times higher probability of being overweight or obese (OR = 2.07, 95% CI: 1.54, 2.78). Neuroticism was associated with a 0.96 times lower probability of being overweight or obese (OR = 0.96, 95% CI: 0.94, 0.98) (see Table 3).

## 4. Discussion

This study attempted to identify factors influencing overweight and obesity in later school-aged children. At the time of the 2019 survey, approximately one-fourth of fifth-grade elementary school children were overweight or obese, with 13.9% classified as overweight and 10.4% as obese. Results from the Korean Student Health Examination Sample Statistics conducted on 39,063 students in 2019 showed an obesity rate (including overweight and obesity) of 24.8% (11.1% overweight, 13.7% obese), similar to our study’s findings. However, there were more obese children than overweight children, indicating a different trend. Particularly, the obesity rate among fifth-grade elementary school children has been steadily increasing, from 25.2% (10.8%, 14.4%) in 2018 to 26.6% (12.0%, 14.7%) in 2019, and 34.1% (15.2%, 18.9%) in 2022, with a sharp increase in the obesity rate due to the COVID-19 pandemic [6,45].

According to the World Obesity Atlas 2023 report [46], every country worldwide is affected by obesity, and no country has reported a decrease in the obesity prevalence across the entire population. Examining the obesity rates among 5–19-year-olds by region in 2020, there were variations across regions: in the African Region, it was 3% for boys and 5% for girls; in the Americas, it was 20% for boys and 18% for girls; in the Eastern Mediterranean Region, it was 11% for boys and 11% for girls; in the European Region, it was 13% for boys and 8% for girls; in the South East Asian Region, it was 5% for boys and 3% for girls; and in the Western Pacific Region, it was 19% for boys and 9% for girls. This report predicts a steeper increase in obesity prevalence among children and adolescents, expecting global rates to rise from 10% to 20% for boys and from 8% to 18% for girls from 2020 to 2035. South Korea was found to have a very high annual increase in child obesity of 3.8% over the period 2020–2035, underscoring the need for more proactive and systematic prevention measures for overweight and obese children.

In this study, firstly, the child’s gender emerged as a predictor of overweight and obesity. Girls had a 1.66 times higher probability of being overweight or obese compared to boys. This contrasts with findings from studies conducted on Korean school health examinations [6] and globally, excluding the African Region [46], where boys were reported to be more obese than girls. This discrepancy is likely influenced by sample characteristics such as the proportion of females or obese participants. From early childhood to late childhood, changes in weight affect self-esteem and school adjustment differently between boys and girls [47], and given that girls experience higher stress related to body image than boys [48], gender differences need to be considered in childhood obesity prevention and management. Particularly, late childhood includes girls who start puberty earlier than boys, with the average onset of puberty for girls being 12.8 years old [49]. Therefore, it is necessary to assess the normal range based on physical growth during puberty and one’s own height and weight to determine whether there is overweight or obesity and to provide guidance for individuals to manage their health accordingly.

Secondly, in terms of lifestyle habits, media usage time emerged as a predictor of overweight and obesity. This study supports the findings of a systematic review and meta-analysis [50] reporting a positive association between screen time and obesity among children, without evidence of a non-linear association. The increase in media usage reduces physical activity levels, which subsequently leads to obesity [17]. The Canadian 24 h movement guidelines for children and youths [51] emphasise the importance of not only engaging in various physical activities but also limiting sedentary behavior, particularly screen time. Specifically, regarding physical activity, it recommends engaging in moderate to vigorous activity for at least 60 min on at least 3 days a week. However, factors such as physical activity, dietary habits, and sleep duration, identified as causes of childhood obesity by the Centers for Disease Control (CDC) Healthy Schools [52], did not appear as predictive factors for overweight and obesity in late childhood in this study. Physical activity was assessed through self-report surveys rather than objective measurements such as direct observation or wearable technology, which could have influenced the research outcomes. Self-reported data on physical activity may suffer from reduced reliability due to factors such as recall bias and individuals’ tendencies to overestimate or underestimate their activity levels [53]. Additionally, a prospective cohort study investigating predictors of overweight/obesity and changes in body mass index (BMI) in 2755 Irish children aged 6–10 years [16] found that the effectiveness of healthy lifestyle behaviours varied depending on the timing of BMI measurements. Children exhibiting healthier lifestyle behaviours experienced reduced risks of overweight/obesity during follow-up observations, emphasising the need to consider not only adopting but also maintaining a healthy lifestyle. Another study [20] showed that lifestyle interventions had only a slight effect on weight reduction in severely obese children, and interventions focusing on individual behaviour changes such as increasing daily physical activity or optimising diet had limited effectiveness globally and could not curb the rising obesity rates [54]. Therefore, in addition to limiting media usage time and engaging in at least 60 min of moderate to vigorous physical activity on at least 3 days a week, increasing daily physical activity in schools, community-based and environmentally oriented measures such as prohibiting advertisements for unhealthy foods or imposing taxes, encouraging healthy food choices, and establishing mandatory standards for school meals are necessary to promote health.

Thirdly, in terms of psychosocial characteristics, both body esteem and Neo personality emerged as predictors of overweight and obesity. This aligns with the findings suggesting that late childhood individuals who perceive their body image as overweight and obese are more likely to be obese [31], supporting the results of this study. Body esteem represents a mental representation of the body formed through one’s bodily experiences and interactions with the environment [55]. In this context, societal pressure for thinness during childhood experiences leads to distorted body images [56]. Such distorted attitudes towards weight and obesity were found to be riskier, with increasing levels of overweight, female students, binge-eating habits, and earlier onset of obesity [57]. Especially within the influence of media preferences for thin bodies, obese children in societies where obesity is socially stigmatised may experience lower self-esteem and negative effects on both physical and emotional aspects [55,58,59].

Among the NEO five personality traits, neuroticism emerged as a predictor of overweight and obesity. This differs from previous research findings that showed higher levels of neuroticism in obese children compared to normal-weight children [60]. In the case of Korean adult women, neuroticism was found to be inversely associated with BMI (β = −0.026, *p* < 0.05), which is similar to the results of this study where there were more female students [34]. Personality traits consistently showed associations with body mass index (BMI), although there was considerable heterogeneity in associations across studies [61,62,63]. The association between personality and BMI varied depending on the factors, indicating that personality–BMI associations are facet-specific [63]. Therefore, the findings of this study examining the relationship between overweight/obesity and personality traits in later school-aged children are thought to be helpful in explaining obesity-related behaviours and providing information for prevention and intervention plans.

The strength of this study lies in identifying psychosocial characteristics as predictors of overweight and obesity in late childhood. Peer acceptance is important for children in late childhood, and feeling different from or being bullied by peers can have lifelong effects on the child [49]. Most obesity programmes conducted in schools focus on lifestyle habits without considering individual characteristics, leading to potential failure in weight management or regaining weight after successful weight loss [64]. Recent interventions for childhood obesity have combined lifestyle improvement with cognitive–behavioural therapy, which has been shown to be more effective in maintaining weight loss and effectively treating psychosocial issues such as body image distortion and depression compared to lifestyle interventions alone [24]. This study has identified not only lifestyle factors but also psychosocial factors such as body esteem and neurotic personality traits as predictors of overweight and obesity in later school-aged children. Research on obesity in late childhood can serve as evidence for proactive early intervention, recognising that obesity is not just a childhood issue but can extend into adolescence and adulthood [65]. Therefore, interventions for overweight and obesity in late childhood should consider not only lifestyle factors but also psychosocial characteristics such as body image and personality traits on an individualised basis for each student. Furthermore, the strengths of this study include high internal and external validity due to a representative sample and rigorous sampling procedures. Trained interviewers (with over 90% having prior survey experience) collected data through face-to-face interviews using TAPI from 2008-born newborns sampled from medical institutions nationwide in Korea, reducing sampling bias. Data cleaning is also conducted to minimise nonsampling errors.

## 5. Limitations

This study is based on data collected before the COVID-19 pandemic. The pandemic has brought about significant changes, including increased overweight and obesity rates compared to pre-pandemic levels. Factors such as unhealthy food choices (e.g., potatoes, meat, and sugary drinks), reduced physical activity due to indoor restrictions, and sedentary lifestyles [13,14] have contributed to this trend. Additionally, there have been notable shifts in mental health, with increased psychological stress and depression [66]. While this study provides insight into the psychosocial characteristics of overweight and obese children in late childhood, its scope is constrained by the fact that the data predates the COVID-19 pandemic. Specifically, the impact of the pandemic on school-aged children and their psychosocial factors limits our ability to fully comprehend its effects. Therefore, further research is needed to compare predictors of overweight and obesity in school-aged children before and after the COVID-19 pandemic.

Another limitation lies in the vulnerability associated with the tools used to measure lifestyle factors, which are considered major influencers of obesity. Most of the instruments used for measuring lifestyle factors in this study, being secondary analysis research, are composed of single questions. Specifically, guidelines, both domestic and international, recommend moderate to vigorous physical activity for at least 60 min a day, a minimum of three days a week, for school-aged children [40,51]. However, PSKC suggests a minimum of only 30 min a day, indicating a need for improvement. Additionally, incorporating more direct measurement instruments such as accelerometry or even validated pictographic questionnaires for the age groups under study is warranted. Furthermore, many tools rely on subjective assessments from both children and parents, often lacking objective measures such as accelerometer data for physical activity. This reliance on questionnaires poses vulnerabilities associated with forgetfulness, thus requiring caution in interpretation. Additionally, the cross-sectional design of the study limits its ability to account for differences in maturity reflected in the secondary sexual characteristics of late childhood between boys and girls, as well as individual variations in growth and development, which can affect BMI in children.

## 6. Conclusions

This study identified factors influencing overweight and obesity in late childhood. The higher the body esteem score, the greater the likelihood of being overweight or obese, along with being female, increasing media usage time, and having lower neuroticism scores. Therefore, to prevent overweight and obesity in late childhood, multidimensional and comprehensive strategies that include behavioural, psychological, and environmental risk factors need to be developed. To effectively combat the prevalence of overweight and obesity in late childhood, interventions must be tailored to address the diverse range of risk factors involved. This necessitates collaboration among healthcare professionals, policymakers, educators, and community stakeholders to develop comprehensive, evidence-based strategies.

## Figures and Tables

**Table 1:** Participants’ characteristics (n = 1174).

Variables	Categories	n (%) or M ± SD	Range (Min–Max)
Obesity level	Normal weight	889 (75.7)	
	Overweight	163 (13.9)	
	Obesity	122 (10.4)	
Gender	Male	609 (51.9)	
	Female	565 (48.1)	
Subjective socioeconomic status		6.92 ± 1.71	1.0–10.0
Health status		4.19 ± 0.69	1.0–5.0
Healthy eating (10 items)		1.91 ± 0.31	1.1–2.9
Physical activity (≥30 min, days per week)	None	330 (28.1)	
	1~2	389 (33.1)	
	≥3	455 (38.8)	
Media usage time (hrs./day)		1.78 ± 1.01	0.0–6.0
Sleep duration (average hrs. per weekday)		8.93 ± 0.66	7.0–12.0
Body esteem (5 items)		1.90 ± 0.53	1.0–3.8
NEO Personality			
Neuroticism		47.67 ± 8.55	29.0–81.0
Extroversion		51.61 ± 9.39	25.0–79.0
Openness		47.16 ± 9.44	10.0–79.0
Agreeableness		51.97 ± 9.16	21.0–84.0
Consciousness		47.09 ± 8.67	20.0–75.0

hrs. = hours; M = mean; Max, maximum; Min, minimum; SD = standard deviation.

**Table 2:** Difference in overweight and obesity according to participants’ characteristics (n = 1174).

Characteristics	Categories	Normal (n = 889)	Overweight and Obesity (n = 285)	χ^2^ or t *(p)*
		n (%) or M ± SD	n (%) or M ± SD	
General factors				
Gender	Male	451 (50.7)	114 (40.0)	9.955 (0.002)
	Female	438 (49.3)	171 (60.0)	
Subjective socioeconomic status		6.95 ± 1.71	6.84 ± 1.71	0.954 (0.341)
Health status		4.18 ± 0.69	4.22 ± 0.70	−0.848 (0.397)
Lifestyle behaviours				
Healthy eating		1.91 ± 0.31	1.92 ± 0.31	−0.169 (0.866)
Physical activity (≥30 min, days per week)	None	256 (28.8)	74 (26.0)	0.897 (0.639)
	1~2	293 (33.0)	96 (33.7)	
	≥3	340 (38.2)	115 (40.4)	
Media usage time (hrs./day)		1.72 ± 0.99	1.95 ± 1.07	−3.330 (0.001)
Sleep duration (average hrs. per weekday)		8.94 ± 0.66	8.88 ± 0.66	1.366 (0.172)
Psychosocial factors				
Body esteem		1.87 ± 0.52	2.02 ± 0.52	−4.322 (<0.001)
NEO personality				
Neuroticism		47.76 ± 8.51	47.37 ± 8.66	0.677 (0.498)
Extroversion		51.95 ± 9.26	50.55 ± 9.73	2.181 (0.029)
Openness		47.28 ± 9.47	46.80 ± 9.35	0.744 (0.457)
Agreeableness		52.10 ± 8.95	51.57 ± 9.80	0.843 (0.399)
Consciousness		47.34 ± 8.58	46.31 ± 8.92	1.748 (0.081)

hrs. = hours; M = mean; SD = standard deviation.

**Table 3:** Multiple logistic regression for predictors of overweight and obesity among later school-aged children (n = 1174).

Variables	Odds Ratio	95% CI	*p*
Gender(ref. = male)	1.66	1.24, 2.21	0.001
Subjective socioeconomic status (per 1 point)	0.98	0.90, 1.06	0.613
Health status(per 1 point)	1.16	0.95, 1.43	0.154
Healthy eating (per 1 point)	0.85	0.54, 1.36	0.503
Physical activity (≥30 min, days per week)	1.05	0.88, 1.25	0.573
Media usage time (per 1 h)	1.23	1.07, 1.41	0.003
Sleep duration (per 1 h)	0.93	0.75, 1.15	0.490
Body esteem (per 1 point)	2.07	1.54, 2.78	<0.001
NEO Personality			
Neuroticism	0.96	0.94, 0.98	0.001
Extroversion	0.99	0.97, 1.01	0.230
Openness	1.02	1.00, 1.04	0.095
Agreeableness	0.99	0.97, 1.02	0.440
Consciousness	0.98	0.96, 1.01	0.161
−2 log 1243.223, χ^2^ = 57.133, *p* < 0.001, nagelkerke 0.072			

CI = confidence interval; ref. = reference.

## Data Availability

Data are available upon request due to ethical and privacy restrictions.
